#  Prevalence and Risk Factors of Hypertension among Male Occupational Bus Drivers in North Kerala, South India: A Cross-Sectional Study

**DOI:** 10.1155/2014/318532

**Published:** 2014-04-23

**Authors:** Arjun Lakshman, Neeraj Manikath, Asma Rahim, V. P. Anilakumari

**Affiliations:** ^1^Govt. Medical College, Kozhikode, Kerala 673008, India; ^2^Department of Internal Medicine, Medical College, Kozhikode, Kerala 673008, India; ^3^Department of Community Medicine, Medical College, Kozhikode, Kerala 673008, India; ^4^Department of Nuclear Medicine, Medical College, Kozhikode, Kerala 673008, India

## Abstract

*Background*. Hypertension is a leading cause of morbidity and mortality worldwide. We aimed to evaluate the prevalence of hypertension in a population of male bus drivers in North Kerala, India. *Methods*. The study population included male bus drivers of Corporation Bus stand Kozhikode, Kerala. Blood pressure, height, and weight of subjects were measured, and relevance was obtained using a structured questionnaire. *Results*. Age varied from 21 to 60 years (mean 36.5 ± 8.4). Among 179 bus drivers studied, 16.8% (30/179) had normal BP, 41.9% (75/179) had prehypertension, and 41.3% (74/179) had hypertension. Isolated systolic HTN was seen in 6.70% (12/179) individuals. Out of 74 hypertensives, 9 (12.1%) were aware of their hypertension, while 3 (4.0%) were medicated and only 1 (1.3%) had BP adequately controlled. Age > 35 years (*P* = 0.015), BMI ≥ 23 kg/m^2^ (*P* = 0.007), supporting more than four family members (*P* = 0.011), and taking main meals from restaurants on most working days (*P* = 0.017) were independently associated with HTN in binary logistic regression. *Conclusion*. Prevalence of hypertension was high among bus drivers. Age > 35 years, elevated BMI, supporting a large family, and dietary habits associated with the job showed significant association with hypertension. Primary and secondary prevention strategies need to be emphasized in this occupational group.

## 1. Introduction

Cardiovascular diseases (CVDs) account for nearly a third of all deaths worldwide. The prevalence of CVDs is increasing in developing countries like India. CVD is predicted to become the major cause of morbidity and mortality by 2020 [1]. The key intervention in CVDs is to identify risk factors early and initiate therapy to control them. An important modifiable risk factor for CVDs is systemic arterial hypertension (HTN). Hence, diagnosis of hypertension and appropriate treatment to optimize BP are important public health goals worldwide.

All groups of professional drivers especially those carrying passengers are at excess risk of HTN, myocardial infarction, and hemorrhagic stroke [2–6]. Occupational bus drivers in a developing country like India deserve special concern. They have to be extremely careful in handling heavy vehicles laden with passengers. Traffic congestion, exposure to vehicle exhausts, constant whole body vibration, poor condition of roads, poor town planning and traffic regulation, over speeding due to competition between buses, and carelessness of pedestrians contribute to their misery. Besides, most of the drivers are in the habit of eating main meals from hotels and consuming snacks (often oily and fried) and fast food items between trips. Many resort to alcohol and smoking to overcome stress. It follows logically that they may have an additional risk of developing HTN. No study has been published regarding the prevalence of HTN or special risk factors among professional bus drivers in India. In this context, we aimed to estimate the prevalence of HTN and to identify its risk factors among male occupational bus drivers of Kozhikode, a city in Kerala, South India.

## 2. Methods

### 2.1. Study Design and Sample

This cross-sectional study was carried out in the Corporation bus Stand, Kozhikode, one of the busiest bus stands in Kerala. The study was conducted during September and October, 2008. The approximate prevalence of HTN among males in Kerala as determined in previous studies is 35% [7–9]. The estimated sample size for our study was 88. But we decided to study all male bus drivers enlisted in the Corporation bus stand giving consent to participate in the study.

### 2.2. Data Collection and Measurements

179 drivers participated in the study. Those who reported violent physical exercise, smoking, tobacco chewing, or drinking tea or coffee within thirty minutes prior to BP measurement were excluded. Information about their medical history, lifestyle, and occupation was obtained by personal interview using a pretested semistructured questionnaire. BP was measured manually using a mercury column sphygmomanometer and stethoscope by the auscultatory method [10]. The subject was seated comfortably for at least 15 minutes before BP recording. Two readings were taken one before and one after the interview, and the two were at least 15 minutes apart. The average of two readings corrected to nearest integer was taken as the BP. Patients with BP in prehypertensive and hypertensive range were asked to report one week later and another set of readings was obtained for confirmation. The lower of the two values was taken as the BP of the individual. We measured the subjects' height and weight, from which the body mass index (BMI) was calculated. The weight was measured (closest to 1 kg) using a beam type weighing scale. Height was measured (nearest to 1 cm) with the subject in an erect position against a vertical surface. Hypertensives were referred for medical care and prehypertensives were advised lifestyle modification and BP monitoring.

### 2.3. Definitions

HTN was defined as systolic BP of ≥140 mm Hg and/or diastolic BP of ≥90 mm Hg or current pharmacological treatment for HTN. Pre-HTN was defined as systolic BP 120–139 mm Hg and/or diastolic BP 80–89 mm Hg. Isolated systolic HTN was defined as systolic BP ≥140 mm Hg in presence of a normal diastolic BP [10].

A subject was said to be “aware” of HTN status if he reported a diagnosis of HTN made by a medical practitioner.

“Treatment for HTN” was defined as regular use of a prescription medication for HTN.

“Control of HTN with treatment” was defined as SBP and DBP less than 140 mm Hg and 90 mm Hg, respectively, in a subject on regular antihypertensive therapy.

The label “never smoked” was assigned to subjects who never smoked cigarettes or beedis and “current smokers” to those who smoked at any time during past one year. “Quit smokers” were those who had quit smoking at least one year before the study. The smoking habit was quantified in pack-years (number of packets of cigarettes smoked per day multiplied by the duration of smoking in years assuming that each packet contains 20 cigarettes).

Based on alcohol use, subjects were classified into “never took alcohol”, “takes alcohol occasionally” (<3 days per week), or “takes alcohol on most days” (≥3 days per week).

### 2.4. Ethical Considerations

Ethical clearance for the study was obtained from the Institutional Ethics Committee, Govt. Medical College, Kozhikode. Informed written consent was obtained from all subjects before including the study.

### 2.5. Statistical Analysis

The collected data was tabulated using Microsoft Excel 2007 and analyzed using EpiInfo 3.5.1. Quantitative variables were summarized as means and qualitative variables were summarized as proportions. Quantitative variables were tested for statistical significance using Student's *t*-test. Qualitative variables were checked for statistical significance using the Chi-Square test. For all statistical tests, a *P* value <0.05 was taken as significant.

## 3. Results

The important sociodemographic characteristics of the study population are summarized in Table 1. Mean age of the population was 36.52 ± 8.38 (SD) years (range 21–60 years). Mean number of family members each subject supported was 4.93 ± 1.71 (range 2–11). Mean number of children per married couple was 1.89 ± 1.03 (range 0–6). Mean number of years for which the study subjects were in the job of bus driving was 14.6 ± 8.27 years (range 1–38). On most days they worked for a mean duration of 11.56 ± 1.87 hours (range 5–16).

The mean SBP and DBP of the study population were 126.66 ±13.74 mm Hg (range 100 to 172) and 83.66 ± 10.18 mm Hg (range 60 to 110), respectively. The mean SBP was 122.19 ± 12.53 mm Hg and 130.25 ± 13.65 mm Hg, respectively, for subjects with age up to 35 years and subjects older than 35 years. Similar values for DBP were 80.45 ± 9.75 mm Hg and 86.25 ± 9.83 mm Hg, respectively. The mean BMI of the study population was 22.77 ± 2.97 kg/m^2^ (range 16.14 to 34.15). The physical examination findings in different age groups are summarized in Table 2.

The systolic and diastolic BPs of study subjects increased with age (for SBP, *r* = 0.306, *P* < .01; for DBP, *r* = 0.289, *P* < .01). The mean values of SBP and DBP were significantly higher in subjects older than 35 years (for SBP, *t* = 4.12, *P* 0.000; for DBP, *t* = 3.94, *P* 0.000).

When the BPs of the subjects were classified according to JNC7 criteria, 16.8% (30/179) (95% CI = 11.2−22.3) had normal BP, 41.9% (75/179) (95% CI = 34.6−49.7) had prehypertension, and 41.3% (74/179) (95% CI = 34.1−49.2) had HTN. Isolated systolic HTN was seen in 6.70% (12/179) (95% CI = 3.4−10.6) individuals. The distribution of the JNC7 categories and isolated systolic HTN in each age group is shown in Table 2.

The association between HTN and various presumed sociodemographic, medical, personal, behavioral, occupational, and dietary risks factors is shown in Tables 3, 4, and 5.

Proportion of the study population belonging to each category of BMI and their distribution according to JNC7 categories of BP are shown in Table 6. Taking the cut-off point for high BMI to be 25 kg/m^2^, hypertension was associated with high BMI (Chi-Square = 5.451; *P* = 0.02). However, on assuming the cutoff point to be 23 kg/m^2^, the level of significance increased (Chi-Square = 8.656; *P* = 0.003).

There was significant association between longer duration of employment as bus driver and hypertensive status. Hypertensives were employed for a mean duration of 16.4 ± 8.22 years, while nonhypertensives were employed for 13.40 ± 8.10 years (Student's *t*-test. *t* = 2.48, *P* = 0.014). In the study population, 7.26% (13/179) (95% CI = 3.46–11.06) were self-reported diabetic patients among whom 61.54% (8/13) (95% CI = 35.09–87.99) were hypertensive also. The mean values of systolic (134.08 ± 18.55 mm Hg) and diastolic (87.54 ± 12.49) BPs were higher in diabetics compared to the remainder. However, no statistically significant association could be discerned between HTN and diabetes mellitus.

Age** >** 35 years, being married, supporting more than 4 family members, taking main meals from restaurants on most working days, eating egg on most days, BMI ≥ 23 kg/m^2^, and longer duration of employment as bus driver were strongly associated with HTN on bivariate analysis. Binary logistic regression was done with these risk factors and current smoking and being diabetic as independent variables (due to strong epidemiological association with HTN in previous studies) as independent variables and HTN as dependant variable. Age > 35 years (*P* = 0.015), BMI ≥ 23 kg/m^2^ (*P* = 0.007), supporting more than four family members (*P* = 0.011), and taking main meals from restaurants on most working days (*P* = 0.017) were independently associated with HTN.

Out of 74 hypertensive individuals, 12.16% (9/74) (95% CI = 4.72−19.6) were aware of their hypertensive status, while only 4.05% (3/74) (95% CI = 0.00−8.54) were treated and only 1.35% (1/74) (95% CI = 0.00−3.98) had BP adequately controlled. No significant association was found between awareness about HTN and educational qualification (Chi-Square-0.033, *P* = 1.000).

## 4. Discussion

Through our study we aimed to report the prevalence and special risk factors of HTN among occupational bus drivers, a particularly vulnerable group. 41.3% of the study population was found to be hypertensive. The only other study on transit vehicle operators in India deals with HTN among autorickshaw drivers of Nagpur among whom the reported prevalence was 35.14% [11]. Only two studies have been published from South Asia on HTN in drivers. In one study, 200 male bus drivers working for Bangkok Mass Transit Authority were screened for HTN. 23% of bus drivers were found to be hypertensive compared to 15.85% of the normal population [12]. Another study conducted by Saleekul et al. in Bangkok observed the prevalence of diastolic HTN to be 17.5% against 7.7% of controls [13]. But numerous studies have investigated this problem in the West. HTN rates among drivers were higher than rates in comparison groups [14]. Ambulatory BP in drivers before, during, and after driving shifts was high [15]. Professional drivers especially those carrying passengers were found to be at an increased risk of stroke, attributed to HTN and stress [6]. A survey showed that 74% of Irish taxi drivers were hypertensive [16].

The overall prevalence of HTN in the study group is much higher than the reported pooled prevalence of about 16–20% in India [17–19]. In the general population also, the prevalence of HTN is higher in Kerala compared to the rest of India. This has been attributed in previous studies to an advanced state of epidemiological transition existing in Kerala [7–9].

Thankappan et al. reported a HTN prevalence of about 30% among males aged 30–59 years in Kumarakom, a relatively rural area in Kerala [7]. The prevalence of hypertension in similar age group in our study was 47.86%. Another study conducted in Chemmaruthy, Varkala, a rural area, gives HTN prevalence of 31.2% among males 20–59 years old [9]. A third study reports a prevalence of 56.3% among urban males aged 40–60 years which is comparable to our finding of 54.5% in the same age group [8]. This shows that prevalence of HTN among bus drivers is higher than the rural population in Kerala but similar to that observed in urban population of comparable age. 94% of the drivers studied reside in rural areas. The near urban prevalence of HTN in them could be due to the drivers acquiring life style risk factors seen in urban population. This may be due to the habits associated with the job.

Among subjects with age up to 35 years, 21 (26.3%) were hypertensive, which is also high. The genetic predisposition among Indians for acquiring cardiovascular risk factors early in life, coupled with the unhealthy lifestyle, could be the cause of high HTN prevalence in the young [20, 21].

Isolated systolic HTN was detected in 6.7% individuals. JNC7 suggests that poor SBP control is one factor responsible for the unacceptably low rates of overall BP control [10]. Control of isolated systolic HTN can reduce cardiovascular mortality and stroke.

41.9% bus drivers had BP in prehypertensive range. The proportion of prehypertensives in a middle aged urban population of Thiruvananthapuram was about 36%. The study from Kumarakom reported pre-HTN rate of 48.3%, while only 15.3% had normal BP. In our study, only 16.8% of the study population had normal BP. This high degree of morbidity is alarming as prehypertensives could develop HTN later. The same has been proven in a recent study conducted among residents of rural Kerala [22].

Prevalence of HTN increased with age as reported in prior studies. There was strong correlation between age >35 years and hypertensive status. The responsibility of supporting more than four family members was found to be associated with HTN. Supporting a larger family obviously requires more money. The possibility of drivers working overtime to meet their financial needs was considered, but average work hours among the two groups were same. Probably, having to support a large family caused a degree of mental stress and anxiety which contributed to the risk of HTN.

Consuming main meals from restaurants on most days was another factor associated with HTN. The association of HTN with dietary sodium intake and fat content and protective role of fruits and vegetables is known. Most of the bus drivers leave their home early in the morning to start work. There may be practical difficulties in preparing tiffin early in the morning. In spite of a person carrying tiffin, he may not get adequate time, space, or other facilities to enjoy his homely meal, during a short stoppage in a long trip. To circumvent this problem, most drivers are forced to take meals from restaurants, whose food often contains excess salt, oil (the same oil may be used repeatedly for deep frying which is harmful), spices, and other additives to make it more palatable. This may explain the association we report.

There was significant association between prevalence of HTN and being overweight (BMI ≥ 25 kg/m^2^). This is in accordance with the published studies. It is seen that morbidity and mortality due to CVDs in Indians are higher in people with lower BMI when compared to Western population [17]. So, some have advocated a cutoff point of 23 kg/m^2^ for normal BMI among Indian population. In the current study, when the cutoff point for high BMI was made 23 kg/m^2^, the significance levels increased [17]. This supports the proposed cutoff of 23 kg/m^2^.

We found significant association between HTN and duration of employment as bus driver. Nevertheless, it was not an independent predictor in regression analysis. This was probably due to age acting as a confounder. Some of the studies have reported duration of employment as a risk factor [6, 12].

Many studies have attributed the higher prevalence of HTN among bus drivers to job stress especially while working in heavy traffic (a “work barrier” which causes risky behavior or needs extra effort), factors in the occupational environment, erratic work hours, sedentary lifestyle, and a sense of underpayment [2, 4–6, 15]. There are also studies that say that there is no association between stress and HTN, while some others report inverse association between self-reported stress and HTN [19, 23]. In our study, we explored this aspect with self-reported traits like getting irritated often, frequently involving in heated exchanges, rash driving, previous involvement in accidents, and subjective feeling of adequate sleep and exercise. We could not find any association between these factors and HTN. Explanations that have been given for this inconsistent association between stress and HTN include adverse situations making a person angry which facilitates development of HTN and HTN altering perception of the degree of stress [23].

HTN and diabetes mellitus often go hand in hand as part of the metabolic syndrome. However, we could not find any association between the two in the present study. This may be due to the small sample surveyed. In addition, our study population was relatively young, about 70% being below 40 years. Around middle age, many bus drivers tend to leave the vocation. In the young, type 2 diabetes is asymptomatic and often goes undiagnosed unless blood sugar is estimated. Our study relied entirely on subjects' self-reporting of diabetes if previously detected. 61.54% of diabetics were having HTN in our study, a figure comparable to the Kumarakom study [7]. We observed that diabetics have higher mean values of systolic and diastolic BPs.

We analyzed the relation between HTN and (a) smoking status (current smokers versus nonsmokers and quit smokers) as well as (b) degree of exposure estimated as pack-years of smoking. We were not able to find any association between smoking and HTN. There was no difference in distribution of smoking among hypertensives and normotensives. It may be possible to clarify the relation by studying a larger sample of the population.

Consumption of fish on most days was found to be protective against development of HTN in one study, likely explanation being the higher content of polyunsaturated-*ω*3-fatty acids in fish [19]. We could not find such a protective effect in the present study. This could be because, majority of the population under study are habitual fish eaters. (96%), so that the protective effect could not reveal itself for lack of contrast. It could also be due to the culinary practices like taking fried fish.

The levels of awareness, treatment, and adequate control of BP are poor in India compared to the West [10]. In the study from rural Kerala, 20.6% of men were aware of their hypertensive status, 16.4% were treated, and 4.6% had their BP controlled [7]. These figures were 33.7%, 22.8%, and 8%, respectively, among urban males of Kerala [8]. Awareness about HTN (12.1%) among bus drivers is lower than the above values. The proportion of hypertensives receiving treatment and having their BP reduced to target levels is very low. This low level of awareness, treatment, and optimal control of BP is unacceptable considering the high literacy level of Kerala. Poor awareness about HTN may reflect (a) lack of frequent contact with the health system, (b) deficits in routine clinical examination on patients, or (c) a lower degree of overall awareness about CVDs, risk factors, and prevention.

The principal strength of our study was that multiple BP readings were obtained. All the measurements were done in the field. Subjects with abnormal BP were advised regarding appropriate medical care. One of our limitations was not measuring the abdominal circumference which correlates better with HTN than BMI. We did not quantify ingestion of salt and alcohol. Employing a questionnaire which will objectively assess the driving habits and compare the stress levels with other occupations will provide more insight into the factors contributing to HTN. Similar studies should be conducted on a larger scale at multiple centers and involving more refined techniques and expertise to yield better results.

To conclude, the prevalence of HTN among male bus drivers of Kerala is very high. A close-to-urban unhealthy lifestyle acquired as part of their job lies at the heart of the problem. They are at risk of developing coronary artery disease, stroke, and chronic kidney disease in future. This will cause significant mortality and morbidity, reduce economic and social participation, and burden their family as well as the nation's health system. All occupational drivers should be screened for lifestyle diseases at entry into the vocation and then periodically. This by itself can reduce mortality due to CVDs [2]. Those at risk should be rehabilitated to protect their own health and to ensure the safety of passengers. The study underscores the pressing demand for targeted educational interventions, regular health checkups, and appropriate lifestyle modifications among bus drivers.

## Figures and Tables

**Table 1:** Sociodemographic, personal, and occupational characteristics of the study population (*n* = 179).

Characteristic	*n* (%)		
Age (years)			
21–30		52 (29.1%)	
31–40		72 (40.2%)	
41–50		44 (24.6%)	
51–60		11 (6.1%)	
Place of residence			
Rural		169 (94%)	
Urban		10 (6%)	
Educational status			
Illiterate		1 (0.6%)	
Lower primary or functional education		2 (1.1%)	
Upper primary or high school		158 (88.3%)	
Predegree or graduate		18 (10.1%)	
Marital status			
Unmarried		32 (17.39%)	
Married		146 (81.6%)	
Divorced		1 (0.6%)	
Consumption of main meals from restaurants			
Regular		112 (62.6%)	
Occasional		67 (37.4%)	
Number of snacks taken between work hours			
Up to 2		123 (68.1%)	
More than 2		57 (31.9%)	
Dietary habits			
Takes mixed diet		178 (99.4%)	
Vegan		1 (0.6%)	
Consumption of	On most days		Occasionally
Fish	160 (96%)		19 (4%)
Egg	97 (58.2%)		82 (41.2%)
Red meat	91 (54.6%)		88 (45.4%)
Chicken	93 (55.8%)		86 (44.2%)
Oily and fried items	98 (58.8%)		81 (41.2%)
Junk food	52 (31.2%)		127 (68.8%)
Family history of illness			
Hypertension		40 (22.3%)	
Diabetes mellitus		38 (21.2%)	
Renal disease		0 (0%)	
Whether diabetic?			
Yes		13 (7.3%)	
No		166 (92.7)	
Number of family members to support			
Up to 4		86 (48%)	
More than 4		93 (52%)	
Number of children (*n* = 147)			
Up to 2		118 (80%)	
More than 2		29 (20%)	
Addictions			
Smoking			
Non smokers		87 (49%)	
Current smokers		83 (46%)	
Quit smoking		9 (5%)	
Alcohol consumption			
Never consumed alcohol		55 (30.7%)	
Occasionally		107 (86.3%)	
Daily		17 (13.7%)	
Pan or tobacco chewing			
Yes		28 (15.6%)	
No		151 (84.4%)	
Monthly income in rupees			
Up to 4000			
4001–8000		115 (64.3%)	
8001 and above		57 (31.8%)	
Self reported rash driving		7 (3.9%)	
Past history of accidents		53 (29.6%)	
Personal habits		35 (19.6%)	
Gets irritated often			
Sleep inadequate		95 (53.1%)	
Inadequate physical activity		50 (27.9%)	

**Table 2:** Mean values of important physical characteristics and JNC7 categories of BP in age groups.

Age category (years)	Mean SBP (mm Hg)	Mean DBP (mm Hg)	Mean BMI (kg/m^2^)	Non-HTN	Pre-HTN	HTN	Isolated systolic HTN
20–29 (*n* = 39)	119.28 ± 10.48	78.69 ± 8.59	22.00 ± 3.26	13 (33.3%)	19 (48.7%)	7 (17.9%)	2 (5.1%)
30–39 (*n* = 75)	127.49 ± 13.72	84.09 ± 10.57	23.07 ± 3.10	10 (13.3%)	31 (41.3%)	34 (45.3%)	6 (8%)
40–49 (*n* = 52)	129.17 ± 13.43	85.56 ± 9.79	23.16 ± 2.61	6 (11.5%)	21 (40.4%)	25 (48.1%)	3 (5.8%)
50–59 (*n* = 13)	134.00 ± 16.02	88.46 ± 9.34	21.80 ± 2.29	1 (7.7%)	4 (30.8%)	8 (61.5%)	1 (7.7%)
Total (*n* = 179)	126.66 ± 13.74	83.66 ± 10.18	22.77 ± 2.97	**30 (16.8%)**	**75 (41.9%)**	**74 (41.3%)**	**12 (6.7%)**

**Table 3:** Association between sociodemographic and medical characteristics and HTN.

Risk factor	Number of subjects, *n* (%)	Number of hypertensives, *n* (%)	Test statistic (*χ* ^2^)	*P* value
Age				
≤35 years	80 (44.7%)	21 (26.3%)	13.584	**0.000**
>35 years	99 (55.3%)	53 (53.5%)		

Place of residence				
Rural	169 (9404%)	69 (40.8%)	0.327	0.743
Urban	10 (5.6%)	5 (50%)		

Monthly income				
Less than Rs. 4000	74 (41.3%)	35 (39.3%)	1.846	0.114
Rs. 4000 or more	105 (58.7%)	39 (37.1%)		

Educational qualification				
Up to secondary school	161 (89.9%)	67 (41.6%)	0.050	1.000
Higher secondary and above	18 (10.1%)	7 (38.9%)		

Marital status				
Unmarried	32 (17.9%)	7 (21.9%)	6.089	**0.010**
Married or divorced	147 (82.1%)	67 (45.6%)		

Number of family members to support				
Up to 4	86 (48%)	28 (32.6%)	5.265	**0.024**
More than 4	93 (52%)	46 (49.5%)		

Number of children (*n* = 147)				
Up to 2	118 (80.3%)	50 (42.4%)	2.478	0.086
More than 2	29 (19.7%)	17 (58.6%)		

Family history of hypertension				
Present	40 (22.3%)	16 (40.0%)	0.038	0.497
Absent	139 (77.7%)	58 (41.7%)		

Family history of diabetes mellitus				
Present	38 (21.2%)	12 (31.6%)	1.896	0.116
Absent	141 (78.8%)	62 (44.0%)		

Diabetes mellitus				
Present	13 (7.3%)	8 (61.5%)	2.358	0.149
Absent	166 (92.7%)	66 (39.8%)		

**Table 4:** Association between personal and behavioral characteristics and HTN.

Risk factor	Number of subjects, *n* (%)	Number of hypertensives, *n* (%)	Test statistic (*χ* ^2^)	*P* value
Smoking				
Current smokers	83 (46.4%)	37 (44.6%)	0.669	0.449
Never smoked or quit smoking before 1 year	96 (53.6%)	37 (38.5%)		
Current smokers who smoked less than 10 pack-years	61 (73.05%)	24 (39.3%)	2.552	0.137
Current smokers who smoked 10 pack years or more	32 (26.5%)	13 (59.0%)		

Alcohol consumption				
Takes alcohol	124 (69.3%)	56 (45.2%)	2.429	0.140
Does not take alcohol	55 (30.7%)	18 (32.7%)		
Does not take alcohol or takes occasionally	162 (90.5%)	64 (39.5%)	2.429	0.140
Takes alcohol on most days	17 (9.5%)	10 (58.8%)		

Pan or tobacco chewing				
Absent	151 (84.4%)	64 (42.4%)	0.433	0.539
Present	28 (15.6%)	10 (58.8%)		

Gets irritated often				
No	89 (46.9%)	36 (42.9%)	0.150	0.407
Yes	95 (53.1%)	38 (40.0%)		

Sleep				
Adequate	129 (72.1%)	56 (43.4%)	0.816	0.232
Inadequate	50 (27.9%)	18 (36.0%)		

Physical activity				
Adequate	70 (39.1%)	43 (39.4%)	0.411	0.538
Inadequate	109 (60.9%)	31 (44.3%)		

**Table 5:** Association between occupational and dietary characteristics an HTN.

Risk factor	Number of subjects, *n* (%)	Number of hypertensives, *n* (%)	Test statistic (*χ* ^2^)	*P* value
Self-reported rash driving				
No	53 (29.6%)	20 (37.7%)	0.403	0.321
Yes	126 (70.4%)	54 (42.9%)		

Past history of accident				
No	144 (80.4%)	59 (41.0%)	0.041	0.850
Yes	35 (919.6%)	15 (42.9%)		

Taking main meals from restaurants on working days				
Occasionally or never	67 (37.4%)	20 (29.9%)	5.830	**0.019**
On most days	112 (62.6%)	54 (48.2%)		

Snacks taken between working hours				
≤2	122 (68.2%)	48 (39.3%)	0.630	0.515
>2	57 (31.8%)	26 (45.6%)		

Taking fish on most days				
Yes	160 (89.4%)	66 (41.3%)	0.005	1.000
No	19 (10.6%)	8 (42.1%)		

Taking red meat on most days				
Yes	91 (50.8%)	32 (35.2%)	2.911	0.097
No	88 (49.2%)	42 (47.7%)		

Taking egg on most days				
Yes	97 (54.2%)	33 (34.0%)	4.679	**0.034**
No	82 (45.8%)	41 (50.0%)		

Taking chicken on most days				
Yes	93 (52.0%)	35 (19.6%)	1.097	0.362
No	86 (48.0%)	39 (21.8%)		

Taking oily and fried items and junk food on most days				
Yes	98 (54.7%)	40 (40.8%)	0.025	0.880
No	86 (48.0%)	34 (42.0%)		

**Table 6:** Prevalence of hypertension among subjects according to WHO categories of BMI.

WHO category of BMI (kg/m^2^)	Number of subjects, *n* (%)	Normal BP, *n* (%)	Prehypertension, *n* (%)	Hypertension, *n* (%)
<18.5	11 (6.1%)	6 (54.5%)	5 (45.5%)	0 (0%)
18.5–24.99	130 (72.6%)	22 (16.9%)	56 (43.1%)	52 (40.0%)
25–29.99	34 (19.0%)	2 (5.9%)	13 (38.2%)	19 (55.9%)
≥30	4 (2.2%)	0 (0.0%)	1 (25.0%)	3 (75.0%)

BMI: body mass index.
